# Hypoxia in Skin Cancer: Molecular Basis and Clinical Implications

**DOI:** 10.3390/ijms24054430

**Published:** 2023-02-23

**Authors:** Sungmi Jeon, Miyeon Jeon, Sanga Choi, Seongkyeong Yoo, Soohyun Park, Mingyu Lee, Iljin Kim

**Affiliations:** 1 Department of Plastic and Reconstructive Surgery, Seoul National University Hospital, Seoul National University College of Medicine, Seoul 03080, Republic of Korea; 2 Department of Pharmacology and Research Center for Controlling Intercellular Communication, Inha University College of Medicine, Incheon 22212, Republic of Korea; 3 Division of Allergy and Clinical Immunology, Department of Medicine, Brigham and Women’s Hospital, Harvard Medical School, Boston, MA 02115, USA

**Keywords:** skin cancer, hypoxia, hypoxia-inducible factor, cancer reconstruction

## Abstract

Skin cancer is one of the most prevalent cancers in the Caucasian population. In the United States, it is estimated that at least one in five people will develop skin cancer in their lifetime, leading to significant morbidity and a healthcare burden. Skin cancer mainly arises from cells in the epidermal layer of the skin, where oxygen is scarce. There are three main types of skin cancer: malignant melanoma, basal cell carcinoma, and squamous cell carcinoma. Accumulating evidence has revealed a critical role for hypoxia in the development and progression of these dermatologic malignancies. In this review, we discuss the role of hypoxia in treating and reconstructing skin cancers. We will summarize the molecular basis of hypoxia signaling pathways in relation to the major genetic variations of skin cancer.

## 1. Introduction

Intratumoral hypoxia is a common feature of solid malignancies, including skin cancer. Cellular responses to low oxygen tension promote cancer progression by inducing biological processes involved in cancer cell survival, such as angiogenesis and glycolysis. Skin cancer most often arises from cells in the epidermal layer of the skin, which are relatively deprived of oxygen [1]. In this review, we describe the roles of hypoxia and hypoxia signaling molecules in skin cancer cells. The well-established hypoxia-inducible factor (HIF) pathway plays a central role in the hypoxic response of skin cancer cells. In addition, the major genetic alterations of skin cancer and their association with hypoxia signaling are discussed. Furthermore, we summarize the effects of hypoxia on the reconstruction of skin defects after surgical tumor resection.

## 2. Hypoxia and the Skin

Human skin consists of three main layers: the epidermis, dermis, and hypodermis. The epidermis is the outermost layer of the skin that protects the body from foreign substances. The dermis is located below the epidermis and is primarily composed of fibrous connective tissue supporting the skin’s overall structure. It is the thickest layer of skin and contains various dermal appendages, such as hair follicles, sweat glands, and sebaceous glands. The hypodermis (or subcutaneous layer) is the lowermost layer of skin, which primarily consists of loose connective tissue and is a major site for fat storage [2].

**Figure 1:**
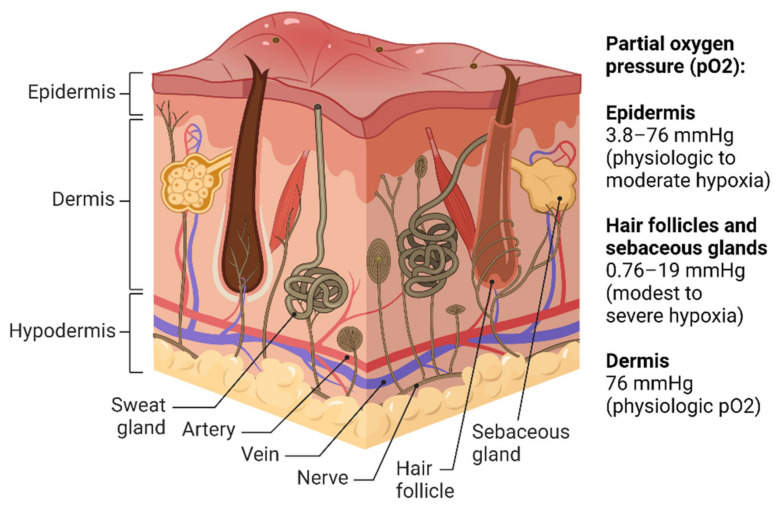
Oxygen levels in different parts of the human skin (created with BioRender.com).

The dermal layer of the skin is well oxygenated, while the epidermis is moderately hypoxic. This is because the dermis receives oxygen directly from numerous blood vessels, whereas oxygen delivery to the epidermis occurs inefficiently via the dermal blood vessels or atmospheric oxygenation of the skin surface. As a result, some skin appendages have a moderate-to-severe hypoxic environment [3] (Figure 1). Although many studies have measured the partial pressure of oxygen (pO_2_) in animal skin, only a few studies have measured it in human skin. The oxygen tension recorded by microelectrodes in the epidermis and dermal papillae of human nail fold skin ranges from 5–25% of the atmospheric value [4]. EF5, a 2-nitroimidazole compound, is a widely used hypoxia marker that selectively binds to cells at low oxygen concentrations [5]. One study that evaluated tissue oxygenation using EF5 values reported physiologic pO_2_ (76 mmHg) in the dermis, physiologic-to-moderate hypoxia (3.8–76 mmHg) in the epidermis, and modest-to-severe hypoxia (0.76–19 mmHg) in hair follicles and sebaceous glands [1].

Skin cancer commonly originates from epidermal cells and later infiltrates deep into the dermis and subcutaneous layers. Three main types of epidermal cells develop into skin cancer: basal cells, squamous cells, and melanocytes. Basal cells lie at the base of the epidermis and gradually rise to the skin’s surface to flatten and become squamous cells, covering the upper part of the epidermis. Melanocytes reside in the basal layer of the epidermis and produce the brown pigment melanin. Melanin protects deeper layers of the skin from DNA damage by blocking ultraviolet (UV) rays. Skin cancer most often results from the abnormal growth of basal cells, squamous cells, or melanocytes [6,7]. Melanoma, which arises from melanocytes, is the most aggressive type of skin cancer worldwide [8,9,10]. Non-melanoma cancers usually refer to basal and squamous cell carcinomas. Nonetheless, there exists rare forms of skin cancer, such as Kaposi’s sarcoma, Merkel cell carcinoma, sebaceous gland carcinoma, and dermatofibrosarcoma protuberans [11].

Hypoxia is a critical feature that promotes the onset and progression of solid cancers [12]. Precancerous skin cells exposed to hypoxic conditions are prone to malignant transformation. In addition, as the tumor mass increases, the oxygen consumption by actively proliferating cells increases, and the oxygen supply decreases owing to insufficient blood vessels, resulting in an imbalance between the oxygen supply and demand. This activates the signaling pathways that promote tumor cell survival and disease progression [13,14].

## 3. Oxygen Sensing and Hypoxia Signaling Pathways

The mechanism of cells adapting to changes in ambient oxygen tension has been described in detail from the 1990s to 2000s. HIF was discovered in 1995 as a transcription factor that binds to the 3′ enhancer element of erythropoietin (EPO) gene [15]. HIF consists of alpha and beta subunits encoded by various genes [16]. HIF-α contains an oxygen-sensitive degradation (ODD) domain targeted by the von Hippel–Lindau protein (VHL) for proteasomal degradation [17,18,19,20]. This degradation occurs only in the presence of molecular oxygen. Prolyl hydroxylases (PHD1-3) use oxygen to attach hydroxyl groups to two conserved proline residues within the ODD domain of HIF-α [21,22,23]. At normal oxygen concentrations, the hydroxylated form of HIF-α is recognized and degraded by VHL.

In contrast, this process does not occur under hypoxic conditions, and stabilized HIF-α relocates to the nucleus. HIF-α forms a dimer with HIF-β (also known as aryl hydrocarbon receptor nuclear translocator, ARNT), which is constitutively expressed in the nucleus and activates the transcription of target genes containing hypoxic response element (HRE) sequences [24]. The transcriptional activity of HIF is regulated by the hydroxylation of an asparagine residue in the C-terminal transactivation domain (CTAD). Factor-inhibiting HIF (FIH), an oxygen-sensitive asparaginyl hydroxylase, prevents HIF from binding to CBP/P300, which are cofactors required for transcriptional activity [25,26]. Three HIF family members are currently known: HIF-1α, HIF-2α, and HIF-3α. Although HIF-1α and HIF-2α share common target genes, each has unique target genes and is known to play independent roles in different cancer types [27,28]. HIF-3α has several variants, with some acting as transcription factors and others as negative regulators of HIF-1/2α [29]. The HIF transcription factors are overexpressed in many solid cancers, including skin cancer, and are linked with a poor patient prognosis [30,31,32]. A sufficient oxygen supply from blood vessels to the skin is restricted to the dermis and subcutaneous layer, and the hypoxic microenvironment of the epidermis can lead to the activation of HIF and other hypoxia pathways [1,33,34,35,36]. The following sections discuss the molecular and cellular mechanisms associated with hypoxia for each type of skin cancer.

## 4. Genetic and Molecular Basis of Hypoxia Signaling in Skin Cancer

### 4.1. Melanomas

Melanomas usually develop in the skin (cutaneous melanomas), but can also occur in the eye (ocular melanomas) and the tissues lining the internal surface of body organs (mucosal melanomas). Cutaneous melanomas account for more than 90% of all melanoma cases in the United States and the majority (75%) of skin cancer-related deaths [37,38]. It is characterized by distinct genetic alterations resulting from mutations that are predominantly induced by UV radiation. Prolonged sun exposure is the most important risk factor for melanomas, and people with pale skin are more susceptible than others. UV radiation causes DNA damage in the epidermis and transforms the melanocytes into melanomas.

Sporadic mutations cause melanomas in approximately 90% of patients. A large fraction of melanoma mutations are UV-induced cytosine-to-thymine transitions at dipyrimidine sites [39]. The most commonly mutated driver genes in cutaneous melanomas are the proto-oncogene B-Raf (BRAF) (~45–50%), Ras GTPase (RAS) (~30%), and neurofibromin 1 (NF1) (~10–15%). These mutations usually occur during the early stages of tumor development. A cutaneous melanoma lacking the above three mutations is classified as a triple wildtype (~10–15%) [40,41,42]. Other recurrently mutated genes in cutaneous melanomas include the proto-oncogene c-Kit (KIT), telomerase reverse transcriptase (TERT), Rac family small GTPase 1 (Rac1), phosphatase and tensin homolog (PTEN), and tumor protein p53 (TP53) [42,43,44].

**Figure 2:**
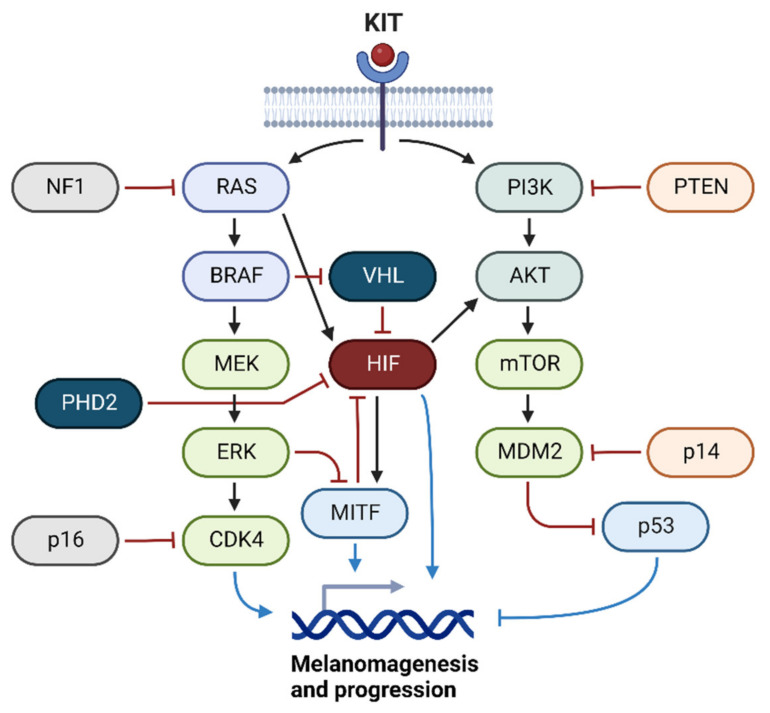
Signaling pathways of melanoma cells in hypoxia. Activation of HIF, along with signaling pathways frequently altered in melanoma cells, contributes to the development and progression of melanomas (created with BioRender.com). KIT, proto-oncogene c-Kit; NF1, neurofibromin 1; RAS, Ras GTPase; PI3K, phosphoinositide 3-kinase; PTEN, phosphatase and tensin homolog; BRAF, proto-oncogene B-Raf; VHL, von Hippel–Lindau protein; AKT, protein kinase B; MEK, mitogen-activated protein kinase; HIF, hypoxia-inducible factor; mTOR, mammalian target of rapamycin; PHD2, prolyl hydroxylase domain-containing protein 2; ERK, extracellular signal-regulated kinase; MDM2, mouse double minute 2; p14, p14 alternate reading frame; MITF, microphthalmia-associated transcription factor; p16, p16 inhibitor of CDK4; CDK4, cyclin-dependent kinase 4; p53, tumor protein p53.

Hereditary melanomas are uncommon, and only ~10% of melanoma patients have an underlying familial predisposition. Germline mutations in the cyclin-dependent kinase inhibitor 2A (CDKN2A) gene are the most frequent, accounting for approximately 20–40% of familial cases [45]. Although CDKN2A was first identified in 1994, followed by cyclin-dependent kinase 4 (CDK4) in 1996, most familial melanoma genes were only discovered after 2010 when next-generation sequencing technologies were introduced [46,47,48,49]. However, although CDKN2A mutations exist in up to 40% of high-density families, mutations in other inheritable genes have been found in less than 1% of familial melanomas. Microphthalmia-associated transcription factor (MITF) has been reported to be a susceptible gene in melanoma families and the general population [49]. A schematic diagram of the signaling pathways involved in melanomagenesis and its progression under hypoxia is shown in Figure 2.

Some of these alterations in melanomas change the mitochondrial metabolism, leading to tumor progression and resistance to targeted therapies [50]. It is known that hypoxic cancer cells increase glycolysis and decrease oxidative phosphorylation compared to normal cells. However, recent studies have shown that oxidative phosphorylation can be enhanced in certain cancers, such as BRAF-mutant or high-peroxisome proliferator-activated receptor gamma coactivator 1-alpha (PGC-1α) melanomas. It has been shown that the expression levels of oxidative phosphorylation-related proteins are increased in patients with BRAF-mutant skin cancer that is resistant to BRAF inhibitors [51,52,53,54]. Oxidative phosphorylation also plays an important role in metastasis because mitochondrial activity is higher in metastatic cancer than in primary cancer [55,56]. Mitochondrial metabolism can stimulate cancer cell proliferation by altering the activity of transcription factors such as HIF-1α, c-Fos, and c-Jun [57]. Several drugs that inhibit oxidative phosphorylation could potentially be used to target specific melanoma subtypes [51]. Photodynamic therapy (PDT) can cause chemical damage to cancer cells by generating reactive oxygen species (ROS) in the presence of sufficient oxygen. Certain nanomedicine-based therapeutics are being tested as a combination modality to reverse tumor hypoxia and improve PDT efficacy [58,59,60].

#### 4.1.1. BRAF

BRAF encodes the serine/threonine protein kinase B-Raf, which plays a vital role in the mitogen-activated protein kinase (MAPK) pathway. The MAPK pathway, initiated by the binding of extracellular factors to receptor tyrosine kinases (RTKs) in the plasma membrane, regulates various cellular processes involved in melanoma development. RTKs then activate Ras GTPases, which sequentially activate effector proteins such as BRAF and phosphoinositide 3-kinase (PI3K) [61]. Most (~90%) BRAF mutations observed in melanomas are V600E mutations, with the second most frequent mutation being V600K (~5%). BRAF-activating mutations cause constitutive enzyme activation and resistance to the negative feedback regulation [62].

In BRAF V600E-positive melanocytes and melanoma cells, the HIF-1α and VEGF levels were elevated, possibly due to the downregulation of VHL expression by BRAF mutations [63]. Similarly, increased VEGF expression has been observed in patients with papillary thyroid carcinomas (PTCs) with the BRAF V600E mutation [64]. It is likely that HIF-1α regulation by BRAF is not limited to melanomas and is equally present in other cancers with BRAF mutations. The BRAF V600E mutation, at least in part, induces angiogenesis through HIF-1α/VEGF, and the treatment of SK-MEL-28 melanoma cells with the BRAF inhibitor PLX4720 reduces VEGF expression and secretion. In an SK-MEL-28 mouse xenograft model, PLX4720 could successfully abolish tumor hypoxia and necrosis by normalizing tumor vasculature [65]. PHD2, which destabilizes HIF-1α, is downregulated in melanomas. In a transgenic mouse created with the Cre–Lox system, the depletion of *Phd2* and the ectopic expression of *Braf* V600E in melanocytes led to melanoma formation. Metastasis to the regional lymph nodes and survival rates were dramatically reduced in these mice. In melanoma tissues, the loss of *Phd2* increases HIF-1/2α protein levels and target gene expression.

Moreover, the protein kinase B/mammalian target of rapamycin (AKT/mTOR) pathway is activated in part through HIFs, and the pharmacological inhibition of mTOR with rapamycin inhibits melanoma growth in mice [66]. In clinical trials, the BRAF inhibitor vemurafenib (PLX4032) showed promising treatment responses in metastatic melanoma patients with the BRAF V600E mutation [67,68]. However, hypoxia may confer resistance to vemurafenib by activating the hepatocyte growth factor/mesenchymal–epithelial transition factor (HGF/c-Met) pathway in melanoma cells. HIF-1α can induce the expression of HGF and c-Met in various cancer cells [69,70,71]. In melanoma spheroids with hypoxic cores, a concomitant treatment with MSC2156119J, a specific c-Met inhibitor, and vemurafenib reduced the resistance of the melanoma cells to vemurafenib [72]. These studies suggest that blocking BRAF-related pathways may be an effective strategy for treating malignant melanomas in a hypoxic microenvironment. A cDNA microarray analysis of BRAF V600E-positive A2058 melanoma cells cultured under hypoxic conditions showed a differential expression of genes related to the cell cycle and apoptosis [73]. Further studies are needed to elucidate the molecular basis of hypoxia-induced cancer progression in BRAF-mutant melanomas.

#### 4.1.2. RAS and NF1

The Ras family of GTPases is an upstream regulator of BRAF. They are small GTP-binding molecules near the plasma membrane that receive signals from membrane-bound RTKs such as c-Kit. RTKs convert Ras proteins from an inactive GDP-bound to an active GTP-bound form. Conversely, NF1 encodes neurofibromin 1, which promotes GTP hydrolysis and the inactivation of Ras proteins [74]. The oncogenic RAS genes in melanoma cells include NRAS, KRAS, and HRAS. However, mutations in NRAS (~30%), but not KRAS (3%) or HRAS (2%), are common in melanomas. NRAS mutations mainly occur in Q61 and result in the ablation of GTPase activity, leading to the constitutive activation of the protein. Similarly, loss-of-function mutations in NF1 can lead to the sustained activation of Ras proteins [75].

In NRAS-driven melanomas, the overexpression of growth factor receptor-binding protein 2-associated protein 2 (GAB2) has been reported to enhance tumor formation and angiogenesis in vivo. GAB2, in concert with mutant NRAS, promotes angiogenesis by stabilizing HIF-1α at the post-transcriptional level and upregulating VEGF expression under hypoxia-mimicking conditions. In a xenograft mouse model, an intraperitoneal injection of bevacizumab, a humanized anti-VEGF monoclonal antibody, reduced tumor growth [76]. Although the c-Kit mutation is known to activate the PI3K/AKT pathway in melanocytes preferentially, it also strongly activates the Ras/Raf/MEK/ERK cascade under hypoxic conditions. In Melan-a mouse melanocytes, the co-expression of mutant c-Kit and HIF-1α lacking the ODD domain successfully induces oncogenic transformation. The treatment of these cells with imatinib, a c-Kit inhibitor, inhibits the hypoxia-induced proliferation and transformation of melanocytes [77].

The hypoxic regulation of Ras pathway proteins has been reported to some extent in other cell types; however, studies on melanomas are still lacking [78,79,80,81,82]. Interestingly, HRAS and KIT were found to possess putative HRE sequences upstream of their transcriptional start sites [83]. Nonetheless, whether these genes are directly targeted for transcription by HIFs in melanocytes remains unclear. Recently, miRNome and proteome profiling of extracellular vesicles (EVs) isolated from BRAF- or NRAS-mutant melanoma cells cultured under hypoxia revealed distinct changes in several factors that could potentially be tested as biomarkers for melanoma progression.

#### 4.1.3. CDNK2A

CDKN2A encodes two alternative splicing variants, the p16 inhibitor of CDK4 (p16INK4A) and the p14 alternate reading frame (p14ARF), transcribed from different first exons, 1α and 1β, respectively. p16INK4A inhibits CDK4 and CDK6, preventing S-phase entry and cell cycle progression. p14ARF stabilizes the tumor suppressor p53 by inhibiting mouse double minute 2 (MDM2)-dependent p53 degradation. Thus, both p16INK4A and p14ARF mutations found in melanoma patients are mostly loss-of-function mutations that result in cell growth and proliferation. Similarly, CDK4 mutations promote the G1 to S phase transition by preventing the p16INK4A-mediated inhibition of the CDK4 [45,49].

Recently, it has been shown that the concurrent inactivation of the p16INK4A and p14ARF pathways, along with the activation of the PI3K/AKT pathway, can induce melanocyte transformation. Interestingly, the AKT-mediated transformation of melanocytes occurs only in hypoxic environments. In CDKN2A-null mouse melanocytes, AKT cooperates with HIF-1α to induce in vitro melanocyte transformation and in vivo tumor growth. Furthermore, the inhibition of mTOR downstream of AKT with rapamycin significantly reduced HIF-1α activity and tumor growth [35,84]. In another study, PI3K/AKT and HIF-1α activated Notch1 signaling under hypoxia during melanoma development. The chemical and genetic inhibition of Notch1 suppressed tumor growth in a xenograft melanoma model [85]. Targeting the PI3K/AKT and HIF-1α pathways may be effective for treating melanoma patients with CDKN2A mutations.

#### 4.1.4. MITF

Genetic polymorphisms resulting in loss-of-function variants of the melanocortin 1 receptor (MC1R) gene are associated with an increased risk of melanomas. MC1R is a G protein-coupled receptor that recognizes α-melanocyte-stimulating hormone (α-MSH) as a ligand and induces MITF via cyclic adenosine monophosphate (cAMP) signaling [86]. MITF, a melanocyte lineage-specific transcription factor, was initially shown to play an important role in melanogenesis through the transcriptional activation of genes involved in melanocyte differentiation. Thus, by producing melanin, MITF protects skin cells from UV radiation-induced DNA damage. However, further studies have shown that MITF is somatically amplified in ~10% of primary cutaneous melanomas and plays a role in promoting the survival of melanoma cells [49,87]. MITF activates nearly 100 genes involved in cell differentiation, apoptosis, proliferation, migration, metabolism, and senescence [88]. Furthermore, cAMP-mediated MITF expression transcriptionally activates HIF-1α in B16-F10 melanoma cells. In addition, MITF upregulates HIF-1α to enhance melanoma cell survival by directly binding to the HIF-1α promoter [89].

Interestingly, in primary melanocytes and UACC62 melanoma cells, MITF expression has been shown to decrease under hypoxia in an HIF-1α-dependent manner. The basic helix–loop–helix family member E40 (BHLHE40, also known as DEC1), a target of HIF-1α, is recruited to the MITF promoter for the transcriptional repression of MITF under hypoxic conditions. This may be a negative feedback mechanism that attenuates oncogenic MITF signaling in melanomas. However, basal MITF expression remains high in the hypoxic tumor microenvironment because of the amplification of MITF itself or other alterations in its upstream regulators [88,90,91].

### 4.2. Basal Cell Carcinoma

BCC arises from the excessive growth of basal cells in the stratified epithelium and is the most common type of skin cancer, representing approximately 75% of all skin cancers. The incidence of BCC is increasing by up to 10% per year, probably due to an increased life expectancy and UV exposure [92,93,94]. BCC mainly affects the head and neck (approximately 70–80%) but can also affect the trunk and extremities [95]. Basal cell nevus syndrome (BCNS, also known as Gorlin syndrome) is an autosomal dominant inherited disorder characterized by multiple BCCs, odontogenic keratocysts, skeletal abnormalities, calcified falx cerebri, plantar or palmar pits, and other clinical manifestations [96,97]. This genetic predisposition to BCC has led to the identification of protein patched homolog 1 (PTCH1) as a highly altered (~70%) gene in patients with BCC [98,99,100]. Loss-of-function mutations in PTCH1 activate the hedgehog (Hh) signaling pathway for BCC carcinogenesis. Similarly, mutations in other factors that activate the Hh pathway, such as gain-of-function mutations in the smoothened homolog (SMO), have been frequently found in BCC cases [101]. Accordingly, inhibitors of the Hh pathway, such as vismodegib and sonidegib, have been tested in clinical trials and approved for the treatment of advanced BCCs [102,103,104].

An immunohistochemical study showed that HIF-dependent hypoxia signals were activated in BCC and trichoepithelioma, a benign hair follicle tumor resembling BCC’s clinical and histological features. Representative HIF target genes, such as BCL2-interacting protein 3 (BNIP3), carbonic anhydrase IX (CAIX), glucose transporter 1 (GLUT1), and VEGF, were also found to be positively expressed in BCC tissues [105]. In another IHC study, HIF-1α was upregulated in human BCC samples compared to normal epidermal tissues [106]. In an analysis of histologically heterogeneous BCC patient samples, CAIX was highly expressed in more aggressive types (~60%) of BCC compared to low-risk types (~10%) and was associated with poor relapse-free survival. BCC subtypes with high recurrence rates include basosquamous, micronodular, morpheaform, and infiltrative [107]. In addition to these histopathological analyses, studies on the role of hypoxia in BCC pathogenesis are limited. In other cancers, hypoxia activates the Hh pathway via HIF-1α. For example, in pancreatic cancer, HIF-1α activates Hh signaling in a sonic hedgehog (Shh) ligand-dependent or -independent manner. The genetic or pharmacological inhibition of HIF-1α inhibits the activation of Hh signaling, suggesting the potential use of HIF inhibitors in treating BCC [108,109,110].

### 4.3. Cutaneous Squamous Cell Carcinoma

SCC is the second most common skin cancer with an increasing incidence. It arises from squamous cells lining the outermost layer of the skin. Non-invasive squamous cell carcinoma in situ, also known as Bowen’s disease, is an early form of skin cancer. Actinic keratosis is a well-known precursor of SCC. Bowen’s disease and actinic keratosis are considered easily manageable diseases; however, if left untreated, they can eventually progress to invasive SCC [111,112,113,114]. Actinic keratosis progresses to cancer at a rate of 0.025–16% per year, depending on the individual lesion [115,116]. In addition, UV radiation-induced mutations in TP53 are frequently found in patients with actinic keratosis and SCC and are considered an early event in SCC development.

Hypoxia promotes the proliferation and abnormal differentiation of skin keratinocytes [117,118,119]. In a K14-HPV16 transgenic mouse model, HIF-1α and its downstream target genes were upregulated during epidermal carcinogenesis [120]. Furthermore, in a UV radiation-induced skin cancer model, an HIF-1α knockout in the epidermis reduced tumor formation and the oncogenic transformation of keratinocytes. Furthermore, the DNA repair ability increased upon UV irradiation in HIF-1α knockout mice compared to that in controls [121]. In keratinocytes, HIF-1α transcriptionally activated several factors related to the nucleotide excision repair (NER) pathway, such as xeroderma pigmentosum (XP) group proteins [122]. Indeed, a double knockout of HIF-1α and XPC resulted in the partial restoration of UV-induced tumor development in HIF-1α knockout mice.

Moreover, in human skin, HIF-1α expression progressively increased during SCC carcinogenesis [121]. In an IHC analysis, HIF-1α and VEGF expressions were consistently higher in SCC than in normal skin or precancerous lesions. In addition, they were associated with an advanced histological grade [123]. Although HIF-1α is generally thought to promote cancer progression, conflicting findings have also been reported. In a transgenic mouse model, HIF-1α gain-of-function suppressed malignant development and the epithelial–mesenchymal transition in squamous cancers [124]. To utilize HIF inhibitors for the treatment of SCC, more preclinical experiments and detailed studies on molecular mechanisms are needed.

### 4.4. Rare Types of Skin Cancer

Kaposi’s sarcoma-associated herpesvirus (KSHV) is an oncovirus that causes Kaposi’s sarcoma, a cancer that usually develops in the skin and mouth of immunocompromised individuals. A KSHV infection induces lytic viral replication by upregulating HIF-1α and HIF-2α [125,126,127]. KSHV upregulates HIF-2α in the endoplasmic reticulum (ER) to enhance the eukaryotic translation initiation factor 4E family member 2 (eIF4E2)-mediated translation of sarcomagenic proteins [126]. In addition, HIF-2α is known to play an essential role as a member of the hypoxia-regulated eIF4FH translation–initiation complex and its role as a transcription factor [128,129]. RNA sequencing of KSHV-infected cells revealed a 34% overlap in the gene expression signatures between KSHV infections and hypoxia [127].

Merkel cell carcinoma (MCC) is a rare neuroendocrine skin cancer with a high risk of metastasis. It usually appears as a painless nodule on the face, head, or neck in the elderly. Merkel cells are located in the basal epidermal layer and are connected to nerve endings involved in the light touch sensation [130]. In MCC tissue samples, HIF-1α is predominantly expressed at the invading edge of the tumor margin [131]. VEGF2 downstream of HIF was also correlated with MCC tumor size [132]. Consistently, another IHC analysis showed that VEGF-A (91%), VEGF-C (75%), and VEGF-R2 (88%) were highly expressed in MCC patients [133]. Therefore, VEGF-targeting bevacizumab has been proposed as a potential drug for MCC treatment [134].

## 5. Hypoxia and Reconstruction after Skin Cancer Resection

After a surgical excision with a safety margin is performed to completely remove skin cancer, the resulting defect should be reconstructed, depending on its size, extent, and location. When primary closure is impossible, various reconstructive options, such as skin grafts and local or free flaps, exist. According to the reconstructive ladder principles, split or full-thickness skin grafts are considered simple to cover defects without bone, cartilage, or tendon exposure. Regarding functional and esthetic aspects, local flaps usually have a better tissue match than distant donor sites, with a skin color and texture similar to the defects resulting from cancer removal. A free microvascular tissue transfer should be performed if the defect is too large or complex. Regardless of the reconstruction method (repair with either grafts or flaps), tissue necrosis is one of the most common postoperative complications and may require secondary surgery. It is usually caused by insufficient blood perfusion or ischemia-reperfusion injury [135,136,137,138,139].

The ischemic and hypoxic necrosis of the implanted flaps resulting from an insufficient blood supply is a major problem in reconstructive surgery. In a rat pedicle flap model, preconditioning flaps by injecting a plasmid encoding HIF-1α improved tissue viability and reduced the necrotic area formation [140]. Similarly, in an ischemic mouse flap model, the systemic activation of HIF-1α with the prolyl hydroxylase inhibitor dimethyloxalylglycine (DMOG) increased flap survival by increasing angiogenesis and inhibiting cell apoptosis. HIF-1α protein levels and CD31-positive vessels increased in the skin flaps of DMOG-treated mice. Circulating VEGF and other HIF downstream angiogenic factors also increased in the DMOG group. In addition, the heterozygous deletion of HIF-1α in mice reduced the survival of ischemic skin flaps [141]. PHD2 depletion in keratinocytes stimulated wound healing in mouse skin [142]. Likewise, in murine diabetic skin ulcer models, the stabilization of HIF-1α with hydroxylase inhibitors or iron chelators promoted wound healing [143,144,145,146]. HIF-1α combined with basic fibroblast growth factor (bFGF) could improve random skin flap survival by increasing VEGF expression in rats [147]. The adenoviral delivery of VEGF increased the viable, non-necrotic surface area and blood perfusion in mouse skin flaps [148]. Rivastigmine, a cholinesterase inhibitor, enhanced angiogenesis in skin flaps by upregulating the HIF-1α and VEGF expression. In one such study, laser Doppler flowmetry and lead oxide/gelatin X-ray angiography were used to evaluate the increased perfusion of skin flaps after rivastigmine treatment [149]. Taken together, these studies suggest that HIF stabilizers may potentially inhibit the ischemic necrosis of the skin flaps. However, HIF stabilizers should be used with caution, as they may increase the risk of cancer recurrence [150].

Ischemia-reperfusion injury triggers the release of inflammatory cytokines and ROS. HIF-1α has an overall beneficial effect on flap survival through angiogenesis, but is also known to promote ROS damage and apoptosis during ischemia-reperfusion injury [151]. Several studies have shown that the ischemic preconditioning of flaps by brief periods of ischemia followed by reperfusion increases the success rate of flap surgery [152]. In addition, ischemic preconditioning induces adaptation of the flaps to the expected fluctuations in blood supply after transplantation. In a rat flap model, ischemic preconditioning lowered the necrosis rate and increased the skin flaps’ survival [153]. Furthermore, the injection of therapeutic stem cells or extracellular vesicles has shown protective effects against ischemia-reperfusion injury [154,155,156,157,158,159,160]. In addition, hypoxia-stimulated ADSCs increased the survival of ischemic rat skin flaps [161]. Therefore, using hypoxia-enhanced stem cells and extracellular vesicles could be an effective strategy to treat ischemia-reperfusion injury.

## 6. Conclusions

Significant efforts have been made to develop inhibitors of the hypoxia pathway for cancer treatment. For example, the VEGF inhibitor bevacizumab has been approved for numerous cancers and has become one of the primary drugs for targeted cancer therapy [162]. In addition, several HIF inhibitors are undergoing phase II and phase III clinical trials [163]. Recently, the FDA approved the HIF-2α inhibitor belzutifan for treating certain cancers associated with von Hippel–Lindau disease, such as renal cell carcinoma, hemangioblastoma, and pancreatic neuroendocrine tumors [164]. However, HIF and VEGF inhibitors require more mechanistic and clinical studies for their application in skin cancer treatment. Conversely, several potential drugs that stabilize HIF, such as roxadustat, are in clinical trials for the treatment of chronic kidney disease-related anemia [165]. HIF stabilizers have the potential to be used for effective reconstruction after skin cancer removal; however, the risk of cancer recurrence must be considered.

Recently developed, new techniques to reverse tumor hypoxia may be applicable to skin cancer. A treatment combining hypoxia-activated chemotherapy with PDT has potential for use in hypoxic tumors. PDT works by converting tissue oxygen into ROS, and this oxygen-dependent mechanism limits the efficacy of PDT in the hypoxic TME. In a recent study, the combination of the hypoxia-activated prodrug tirapazamine (TPZ) and near-infrared (NIR) light-induced PDT showed synergistic effects against CT26 murine colorectal cancer cells under hypoxic conditions [166]. The currently used immune checkpoint blockades (ICBs) are effective at treating various cancers, but have some limitations, in part because they are antibody-based therapeutics. For example, pembrolizumab, a monoclonal antibody against programmed cell death protein 1 (PD-1), cannot affect the cytoplasmic or nuclear distributed PD-L1 protein. A novel nanoparticle, IR-LND@Alb, that can selectively accumulate in mitochondria has been suggested as a novel strategy to reduce PD-L1 expression. IR-LND@Alb inhibits PD-L1 and mitochondria complexes to reduce endogenous oxygen consumption, thereby alleviating tumor hypoxia. The reversion of hypoxia helps improve the efficacy of various tumor therapies [167]. BSA-MHI148@SRF nanoparticles have been shown to enhance PDT efficacy by inducing tumor reoxygenation [168]. Similarly, MB@Bu@MnO2 nanoparticles reverse tumor hypoxia to reactivate immunotherapy [169]. Engineered microalgae capable of photosynthesis can treat cancer cells that are resistant to radiation and phototherapy by increasing local oxygen levels in the hypoxic TME [170]. Conversely, certain drugs can be used based on their hypoxia-dependent cytotoxic effects. Perfluorocarbon nanoparticles can boost the effect of hypoxia-based agents (HBAs) by sustaining the hypoxic TME [171].

In conclusion, a comprehensive understanding of hypoxia biology is needed to accelerate the development of new therapies for patients with skin cancer.

## Data Availability

Not applicable.
